# A step-wise approach for establishing a multidisciplinary team for the management of tuberous sclerosis complex: a Delphi consensus report

**DOI:** 10.1186/s13023-019-1072-y

**Published:** 2019-04-30

**Authors:** Stéphane Auvin, John J. Bissler, Vincent Cottin, Ayataka Fujimoto, Günther F. L. Hofbauer, Anna C. Jansen, Sergiusz Jóźwiak, Larissa Kerecuk, J. Christopher Kingswood, Romina Moavero, Roser Torra, Vicente Villanueva

**Affiliations:** 1 Service de Neurologie Pédiatrique et des Maladies Métaboliques, APHP, Robert-Debré Children’s Hospital, 48 Boulevard Serurier, 75019, 19 Paris Cedex, France; 2 INSERM U1141, Paris, France; 3 St. Jude Children’s Research Hospital and Le Bonheur Children’s Hospital, 848 Adams Ave, Memphis, TN 38103 USA; 4 Claude Bernard University of Lyon 1, Reference Center for Rare Pulmonary Diseases, Louis Pradel Hospital, UMR754, 69677 Lyon, Cedex France; 5 Seirei Hamamatsu General Hospital, 2 Chome-12-12 Sumiyoshi, Naka Ward, Hamamatsu, Japan; 6 University Hospital Zurich, Rämistrasse 100, 8091 Zurich, Switzerland; 7 UZ Brussel, Vrije Universiteit Brussel, Laarbeeklaan 101, 1090 Brussels, Belgium; 8 Department of Child Neurology, Warsaw Medical University, Banacha 1, 02-097 Warsaw, Poland; 9 Department of Neurology and Epileptology, The Children’s Memorial Health Institute, Warsaw, Poland; 10 Birmingham Women’s and Children’s NHS Foundation Trust, Birmingham, B15 2TG UK; 11 St Georges Hospital, Blackshaw Rd, London, SW17 0QT UK; 12 Tor Vergata University Hospital, Via Columbia, 2, 00133 Rome, Italy; 13 Bambino Gesù Children’s Hospital, IRCCS, Rome, Italy; 14 Fundació Puigvert, Universitat Autónoma Barcelona, REDINREN, Carrer de Cartagena, 340-350, 08025 Barcelona, Spain; 15 Hospital Universitario y Politécnico La Fe, Avinguda de Fernando Abril Martorell, 106, 46026 Valencia, Spain

**Keywords:** Tuberous sclerosis complex, Multidisciplinary care, Multidisciplinary team

## Abstract

**Background:**

Tuberous sclerosis complex (TSC) is a rare autosomal dominant genetic disorder associated with mutations in *TSC1* and *TSC2* genes, upregulation of mammalian target of rapamycin signaling, and subsequent tumor formation in various organs. Due to the many manifestations of TSC and their potential complications, management requires the expertise of multiple medical disciplines. A multidisciplinary care approach is recommended by consensus guidelines. Use of multidisciplinary teams (MDTs) has been shown to be beneficial in treating other complex diseases, such as cancer. In a lifelong disease such as TSC, an MDT may facilitate the transition from pediatric to adult care. However, little guidance exists in the literature regarding how to organize an MDT in TSC.

**Methods:**

To discuss the best approach to assembling an MDT, this project was initiated in October 2017 with a meeting of 12 physicians from various specialties and various countries. Following this first meeting, the experts generated statements on the most important aspects to implement in establishing an MDT for TSC by 3 rounds of selection using a Delphi process via electronic correspondence. Finally, TSC patient advocates reviewed the findings and provided additional insights from a patient perspective.

**Results:**

A 3-step roadmap was recommended, starting with identifying a single individual to begin organizing care (Step 1), then establishing a small core team (Step 2), and finally, establishing a larger multi-disciplinary team (Step 3). Because of the multisystemic nature of TSC, the MDT should include specialists such as a neurologist, a neurosurgeon, a nephrologist, a urologist, a pulmonologist, an ophthalmologist, a cardiologist, a dermatologist, a geneticist, and a psychiatrist/psychologist. The MDT should recommend a care plan for each patient based on the individual’s needs and in consultation with him/her or his/her family. Some of the most important aspects of an MDT that were agreed upon included identifying a case manager to help coordinate care, providing access to health care professionals of varying specialties, and including a lead physician who takes medical responsibility for patients’ overall care.

**Conclusions:**

The results of our consensus provide guidance to support the initiation of an MDT in TSC.

## Background

Tuberous sclerosis complex (TSC) is a rare autosomal dominant genetic disorder occurring in 1 in 6000 live births [1, 2]. It is caused by mutations in either the *TSC1* or *TSC2* gene, which result in upregulation of the mammalian target of rapamycin (mTOR) pathway and subsequent tumor growth in various organs such as the brain, heart, skin, eyes, kidney, lung, and liver [2, 3].

Clinical phenotypes of TSC can vary greatly from patient to patient, ranging from milder (such as a single manifestation of TSC) to more severe disease (such as affecting multiple organs, developing early epilepsy and neurodevelopmental issues) [4]. In addition, many manifestations of TSC have age-dependent expression [2]. These factors together can complicate evaluation and management of TSC.

Because many manifestations can lead to complications and evolve over a patient’s lifetime, the expertise of multiple disciplines is needed to effectively manage patients with TSC. For example, cardiac rhabdomyomas can develop prenatally and are thus one of the earliest detectable signs of TSC [2, 5]. Although they are usually asymptomatic and regress with age, these lesions occasionally cause arrhythmias and lead to ventricular dysfunction and thus may require consultation with a cardiologist [2, 5]. Hypomelanotic macules on the skin can be present at birth, while other cutaneous lesions such as facial angiofibromas and ungual fibromas develop later in life and may require treatment by dermatologists [2, 6]. Because skin lesions are prominent manifestations that can be visible at a young age, dermatologists are also in a position to identify TSC early in the disease course [7].

In addition, brain lesions such as cortical tubers, subependymal nodules, and subependymal giant cell astrocytomas, and seizures (which are frequently difficult to treat) can also develop early in a patient’s life and may require input from neurologists, neurosurgeons, and epileptologists [2, 5, 8]. TSC is associated with a wide range of behavioral, psychiatric, intellectual, learning, neuropsychological, and psychosocial difficulties, which manifest in childhood and persist throughout life. These TSC-associated neuropsychiatric disorders (TAND) therefore require regular monitoring and coordination with early intervention and educational specialists, psychologists, psychiatrists, and social workers (childhood to adult specialists) [6, 9–12].

As a patient ages, cysts and angiomyolipomas can develop in the kidneys, which may lead to chronic kidney disease, and thus require the consultation of a nephrologist [2, 5]. Nephrologists may also have an important role managing medications related to the treatment of TSC that require additional monitoring of renal function, such as everolimus [13, 14]. Patients with TSC are also more likely to develop some malignancies, particularly renal cell carcinomas and pancreatic neuroendocrine tumors, and usually at a younger age than the general population [15–17]. Another concern, primarily for female adolescents and adults, is the potential development of lymphangioleiomyomatosis in the lungs, which, if present, requires consultation with a pulmonologist [2, 5].

The 2012 International Tuberous Sclerosis Complex Consensus Conference established recommendations for the diagnosis and treatment of TSC [18, 19]. Because of the complex multisystem nature of the disease, a multidisciplinary approach in the management of TSC was recommended [6, 19–21]. The use of multidisciplinary teams (MDTs) has been shown to be beneficial in improving management and outcomes in patients with other complex diseases [22–26]. For example, multidisciplinary discussion is recommended in the diagnosis of idiopathic pulmonary fibrosis [26]. In a retrospective observational study, MDT discussions were responsible for the definite diagnosis in 80.5% of 938 patients with interstitial lung disease [25]. Of these 938 patients, 455 had received a diagnosis from a referring physician before multidisciplinary discussion, and 41.9% of those patients had their diagnosis changed after multidisciplinary discussion [25]. In addition, diagnosis through multidisciplinary discussion was able to better discriminate between idiopathic pulmonary fibrosis and other interstitial lung diseases compared with diagnoses made before multidisciplinary discussion [25]. In cancer, MDTs resulted in more evidence-based medicine and more timely treatment, and in some cases, led to improved survival [27]. Patient-reported experience of care had the greatest improvement for the particular cancers for which MDTs were more established [27]. In TSC, a multidisciplinary approach is also important in facilitating transition of care from childhood to adulthood as individuals age [20, 21, 28, 29]. Young adult patients have expressed the need for multidisciplinary care that is well informed and easily accessible, focuses on the patient as a whole (including mental and physical health among other factors), and includes his/her family as they transition to adult care [28]. Multidisciplinary care can be useful to reduce the significant burden placed on caregivers of patients with TSC, especially concerning the amount of health care utilization (e.g., doctor visits, procedures, or tests) required to treat the patient [30], as well as on the patients themselves, although further studies are needed to evaluate the benefits of MDT on patient and caregivers.

Some reports have encouraged the use of specialized TSC clinics, which may not be available for all patients because of cost and geography [20]. There are others who have reported their personal experience in running an MDT clinic for TSC, including a list of practitioners involved and how care in their clinic is organized [29]. Other groups have focused on the patient concerns MDTs should consider when implementing a transition of care from youth to adulthood [28]. Still others have performed studies showing how their use of MDT has increased rates of patient follow-up and opportunities for exams or treatments [31]. However, there is still a lack of published literature on how best to approach establishing an MDT. The aim of this report is to announce the results of a task force identifying key steps in developing an MDT in TSC as well as the key components of the development and function of the MDT.

## Methods

A group of 12 physicians from various specialties including neurology (pediatric and adult), psychiatry, TAND, neurosurgery, dermatology, nephrology, and pulmonology convened face to face in October 2017 to discuss the steps needed for organizing an MDT. A 3-step roadmap was agreed upon to help develop an MDT, starting with identifying a single individual to begin organizing care, then establishing a small core team, and finally establishing a larger multidisciplinary team.

In addition to proposing steps for establishing the MDT, the committee of physicians also discussed, based on their expertise, the aspects of the MDT that were most important to implement. These recommendations were produced via a 3-step Delphi process [32] led by one of the authors (SA) via online correspondence. Step 1 required each member to generate a list of 10 statements summarizing key aspects that were important in developing an MDT. For Step 2, all statements were collated, and members were asked to rank statements in the overall list on a scale of 1 to 10 based on importance (1 being least important and 10 being most important), after which the top 16 statements were identified based on those that met a cutoff score of ≥7.5. For Step 3, the top 16 statements were again scored from 1 to 10 by members of the committee, based on priority of implementation (10 being the most urgent and 1 could be implemented later). These 16 statements are discussed in detail further below. The group also discussed the stages of developing a TSC clinic during the formulation of the manuscript.

Input and insight from TSC patients/patient advocates regarding the committee’s findings were sought, which included feedback on existing concepts and additional areas of importance from the patient perspective.

## Results

### Organizing a multidisciplinary care team for the management of TSC

**Fig. 1:**
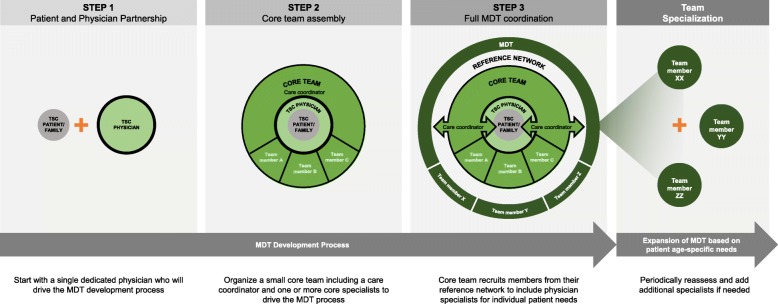
Consensus-established roadmap for development of an MDT

Three steps were identified by the task force/group (Table 1). The authors recommend that throughout this process, input from patients and their families, available research, and sharing of data between specialties should be considered in evaluating how to organize the team (Fig. 1).

**Table 1:** Three-step process in establishing a larger multidisciplinary team

Steps	Participants	Main Action
Step 1	A single physician who has a passion for TSC	• Treat patients with TSC • Liaise with patient families, organizations, and other physicians • Identify a care coordinator • Initial drive of the MDT process
Step 2	Core team (comprising a care coordinator and members of the core medical specialties)	• Liaise with patients/families • Coordinate physicians for management decisions • Organize patient follow-up and referrals • Liaise with other centers • Collaborate with TSC patient organization if available and able
Step 3	Full MDT and reference network with the assistance of the care coordinator	• Operational MDT

*MDT* multidisciplinary team, *TSC* tuberous sclerosis complex

#### Step 1—single person

To establish the MDT, the first step is to start with a single dedicated physician. Ideally, this physician should be able to liaise with the family of the patient and other physicians, nurses, and patient managers, with a shared vision for multidisciplinary care. The physician should connect with all specialists required for patient management in TSC. Patient organizations can assist with identifying physicians interested in developing the MDT team and connecting them with other physicians. An important specialist to include at this stage is a clinical geneticist, who can explain the results of the genetic analysis and provide counselling about risk of inheritance. Because clinical geneticists see both children and adults, in some cases they might be in the position to fulfill the role of the Step 1 physician as well as care coordinator of the MDT.

This initial person does not need to be a senior member of the medical team; however, he or she should have some experience in managing patients with TSC. In addition, as the starting point for the team, this individual should have the energy and stamina to commit to this endeavor and have a sense of personal ownership of the project.

Finally, as information on TSC is ever evolving, the physician should maintain up-to-date knowledge and be able to attend relevant international conferences to better understand the patient perspective and participate in clinical research.

#### Step 2—small Core team

The next step to establishing the MDT is the organization of a small core team, with the goal of obtaining support from the core specialties. Although there is not one set method to establishing the core team, typically the core team should comprise one care coordinator/nurse and one or more physicians (specialists). This core team ensures more continuous connection between the patients/families and the greater TSC team. This allows for easier discussion between physicians regarding the most effective decisions in management and fosters organization of future patient follow-ups.

The core team should help organize appropriate referrals and coordinate scheduling of procedures/office visits and be responsible for identifying contacts at other treatment centers or referral sites, if needed. The core team may further tap into reference networks for other practitioners, focusing on those who specialize in rare diseases.

#### Step 3—multidisciplinary team

To assemble the larger MDT, the core team should recruit members from the reference network they have established. Ideally, an MDT to manage TSC should include physicians with specialties that are required to appropriately address the needs of the patients. These specialties can include pediatrics, genetics, diagnostic radiology, interventional radiology, cardiology, pediatric/adult neurology, respiratory medicine, pediatric/adult nephrology, dermatology, neurosurgery, oncology, fetal medicine, urology, ophthalmology, psychiatry, psychology, intellectual disability psychiatry, and surgery [12, 33]. At this stage, the team should reassess its way of functioning and evaluate whether it adequately meets the needs of the patient community on a regular basis. It is particularly important to establish who is responsible for soliciting this feedback (e.g., the treating physician or nurse).

Ideally, one should begin securing a dedicated care coordinator when putting together the core team; however, a dedicated care coordinator (part-time or full-time, depending on the expected size of the team) is required when the MDT is established. Team members would also benefit from developing leadership and management skills in order to accommodate a plan for shared (or to transfer) leadership as the team grows.

### Most important factors in establishing the multidisciplinary team

The 16 statements produced by the committee regarding the most important factors in establishing the MDT can be found in Table 2, which also includes the mean (standard deviation) score that was calculated for each item at Step 3 of the process described above.

**Table 2:** Top 16 most important aspects to implement in a multidisciplinary TSC team

		Mean Score	SD
1	Identification of a patient care coordinator (nurse or other professional) for duties including case management, answering questions, direct contact with patient, triage for specialists, etc.	9.2	1.1
2	Creation of a list of health care professionals associated with the clinic involved in management of patients with TSC, including their current email, phone number, and availability to see patients	8.7	1.6
3	Identification of a lead physician (with at least 1 or 2 deputies) who takes medical responsibility for the patients’ overall care and managerial responsibility for organizing the clinic	8.4	1.1
4	Organization/identification of care pathway in the clinic and establishing investigation schedule according to patient age (to avoid multiple visits to the hospital)	7.9	1.1
5	Following accepted TSC guidelines/protocol	7.7	2.4
6	Identification of a network of local or regional health care professionals outside of the clinic	7.5	1.2
7	Creation of a plan for diagnosis and management of TSC psychiatric comorbidities (e.g., TAND, autism)	7.4	2.0
8	Establishment of a patient database for case management and research	7.2	1.5
9	Creation of TSC center of excellence for complex cases	6.8	2.6
10	Creation of a plan for transition from pediatric to adult care, including specified age of transition, steps taken, and identification of health care professionals for adult care	6.7	2.4
11	Establishment of MDT meetings that include case discussion, discussion of guidelines, logistics	6.6	1.6
12	Creation of a prenatal diagnosis program (i.e., identifying cardiac tumors)	6.5	2.7
13	Ability to perform investigation imaging such as MRI and CT under sedation (or possibly general anesthesia)	6.4	2.7
14	Establishment of a link between clinic and local patient/family TSC organizations—ideally have a representative of TSC organization present at clinics	6.3	1.7
15	Establishment as a source for dissemination of information on TSC for patients and health care professionals, including any appointments, reports, etc.	6.2	1.1
16	Establishment of communication with patient’s primary care physician and other family physicians to clarify responsibilities	5.9	1.6

*CT* computed tomography, *MDT* multi-disciplinary team, *MRI* magnetic resonance imaging, *SD* standard deviation, *TAND* TSC-associated neuropsychiatric disorder, *TSC* tuberous sclerosis complex

The panelists agreed that identifying a patient care coordinator was the most important aspect of establishing the MDT. An MDT cannot function without organization and cooperation; thus, having a care coordinator allows for the MDT to perform the rest of its functions in providing care efficiently and effectively.

Other highly important aspects of establishing an MDT include creating a list of other health care professionals who would participate in the care of patients with TSC within the clinic. The MDT should have a pool of able health care professionals readily available for patient referral depending on the patient’s needs. A lead physician who is ultimately responsible for patient care and the organization of the clinic should be identified, as well as core physicians in the clinic who may be needed to provide care for the patient. The MDT should identify and communicate with a network of local or regional health care professionals outside of the clinic, including primary care providers and those who treat somatic and behavioral aspects of TSC. Due to the complexity of managing patients with TSC, use of a hub-and-spoke model would assist in keeping primary care physicians involved and informed. Two-way communication between the clinic and the patient’s primary physician is of paramount importance. In some countries the primary physician will be a general practitioner or office generalist doctor, whereas in others it will be a specialist (e.g., pediatrician or neurologist). In addition, establishing a schedule of patient investigation and monitoring is important to avoid compounding the frequency of visits.

A good MDT must also create a plan to handle diagnosis and management of TAND-related conditions. As the majority of patients with TSC will develop some type of neuropsychiatric manifestations across their lifespan, managing these manifestations is critical, especially as TAND is a very significant burden on daily life [9, 34].

Establishing a patient database was also considered to be beneficial to better organize patient care. The patient database can be used for case management and discussion as well as serve as a source for clinical research.

Once established, the MDT can evolve into a TSC Center of Excellence, which is a TSC clinic that provides care coordination, provides many services and can access any others needed, and commits to undertake research by building up experience and keeping abreast of—and ideally participating in—TSC research. Such a center can also exchange experience with other Centers of Excellence. These centers would preferably incorporate spaces where all specialists can be found in one clinic, where the patient can reach each of them easily, or provide a virtual clinic service to simplify patient interaction with specialists. Becoming a Center of Excellence in TSC is ideal, although this may not be feasible in all facilities; nevertheless, the MDT should strive towards this goal.

The team should create a plan for transitioning pediatric patients to adult care. Medical issues and concerns change with age and may pose challenges for patients who transition to more adult-oriented care [6]. Ideally, the process of establishing and building an MDT TSC clinic for adults should be underway at the same time that clinics for children are being established, or the same clinic should be used for both children and adults. Transition to adult care may be especially challenging for individuals with TSC because of TSC-associated intellectual disability and other TAND-related issues. Therefore, the MDT for adults must have familiarity with managing intellectual disability and TAND.

The MDT will also benefit from holding regular meetings. These meetings can increase collaboration and promote discussion of patient cases and current guidelines, [18, 19] and can be used to keep team members up to date on the progress of basic or clinical research, and may be conducted either in the clinic or elsewhere. In addition, meetings provide an opportunity to discuss and align on protocols used in the clinic.

Creation of a prenatal diagnosis program is another important function of the MDT. Cardiac rhabdomyomas are a major feature of TSC and are commonly observed in prenatal ultrasound of patients with TSC (up to 80%) [18, 35]. In addition, brain lesions such as cortical tubers and subependymal nodules are often reported prenatally, and subependymal giant cell astrocytomas that develop in early childhood are other early indicators of TSC [2, 8]. Prenatal diagnosis programs should also include genetic testing, not only to confirm diagnoses but to assess the patient’s potential phenotype [36]. Early identification of patients with TSC offers the opportunity to provide optimal parental education on epilepsy and TAND and to initiate early treatment and interventions with the aim of improving clinical outcomes [37]. For example, early identification and management of seizures in infants with TSC can potentially improve cognitive outcomes [38]. Research into useful approaches to make an early diagnosis before seizure onset has been recently conducted [35].

Young patients with TSC will require routine imaging via magnetic resonance imaging (MRI), so it is important that the MDT has the capability to perform these imaging studies with sedation (possibly general anesthesia in some cases) and, where possible, they should combine imaging studies of multiple areas, such as the brain and abdomen using an adapted imaging protocol [19].

Patient and disease organizations can help in supporting patients and research. It is useful for the MDT to establish a relationship with such organizations, preferably with a representative of a TSC organization participating in their clinics. Close collaboration with the patient organizations throughout can be of benefit at all stages of MDT development, including initial set-up, growth, full expert team, and later can be helpful for regular assessment of further needs. Patient organizations can also give complementary support such as offering social-emotional feedback, giving information on the disease in lay-language, connecting patients with others in similar situations, and providing a feeling of community.

Lastly, it is important for the MDT to serve as a source of information for patients and other health care professionals and to communicate effectively with patients’ primary care physicians.

### TSC clinic development

The development of a TSC clinic can occur in several stages (Table 3). The primary characteristic of a TSC clinic is that it is a service willing to take responsibility for coordinating and ensuring access to systematic and holistic care for patients with TSC and their families. This includes delivering care based on established clinical guidelines. If it is a pediatric-only clinic, an established plan should be in place for the transition of older pediatric patients to adult services. The clinic should comply with good medical practice, ensuring communication of individual patient management plans to all appropriate parties and also disseminating knowledge of TSC and its management in general. Access to MRI and computed tomography scans under sedation is also vital, ideally combining imaging of the brain and abdomen in a single program. In addition, all TSC clinics and TSC centers of excellence should be aware of laboratories in their country where genetic analyses are performed and should be able to request these tests when needed.

**Table 3:** Stages of TSC clinic development

Stage of Clinic Development	Services Provided
Maturity Level 1	• Care coordinator o Schedule appointments and investigations o First point of contact for advice • Lead Clinician o With TSC interest and expertise o Created referral pathways • Close collaboration with patient/family organizations
Maturity Level 2	• Care provided jointly by a core group of TSC-knowledgeable physicians from relevant specialties^a^ • MDTs to review cases and agree on management plans • Facilitate advice and care from a broader network of specialties and from a wider geographic area than the clinic can provide • Establish links with other TSC clinics (via MDTs or other joint meetings for peer-to-peer continuing education) • Formalize surveillance for TAND problems, e.g., using the TAND checklist • Use database to facilitate monitoring of patients
Maturity Level 3	• Work with larger network of local TSC-knowledgeable specialists; within the same care provider or closely allied care providers • Establish and promote local and regional network of specialists and care providers; acting as expert center providing advice and at least annual holistic review • Provide expertise for review of mental health and coordination of referrals for mental health problems
Maturity Level 4 (Center of Excellence)	• All the above, plus engage with research into TSC

*MDT* multi-disciplinary team, *TAND* TSC-associated neuropsychiatric disorder, *TSC* tuberous sclerosis complex

^a^Specialties useful in providing TSC services: pediatrics, genetics, diagnostic radiology, interventional radiology, cardiology, pediatric/adult neurology, respiratory medicine, pediatric/adult nephrology, dermatology, neurosurgery, oncology, fetal medicine, urology, ophthalmology, psychiatry, psychology, intellectual disability specialists, surgery

### Patient perspective

The MDT provides patients with a team-based approach to care decisions. Without the MDT, patients may end up receiving a variety of differing medical opinions, a very challenging sequence of regular examinations, and medications prescribed by different specialists. Patients appreciate that the MDT provides them with a single treatment plan so that medical advice is not contradictory. To this end, patients emphasize the importance of having a single care coordinator or an individual who is responsible within the group of health care professionals. The level of communication with the patient’s other health care providers is also important. In addition, patients appreciate the increased organization of care that comes with the MDT, such as combined imaging studies, and in general, prefer the holistic approach to treatment that the MDT offers (education, social support, etc.). In general, future studies should focus on assessing the benefits of MDT on patient care, including what aspects of the MDT are most important and what works best.

TAND is an especially important concern of TSC to patients/caregivers. Although not generally life-threatening, TAND represents the most significant burden on daily life experienced by patients with TSC, and as such, it has not been assessed and addressed sufficiently. A committed MDT should undertake a systematic evaluation of TAND, facilitate prevention of TANDs, and have a plan in place for crisis intervention.

Patient organizations should have an integral role in the formation of the MDT. There are numerous possibilities for collaboration between these organizations and the MDT, such as helping patients find the right kind of social support, education, family support, leisure activities, and so on. Patient organizations can also help in finding the best clinicians with a passion for taking care of patients with TSC.

## Discussion

Based on the discussions of our multidisciplinary consensus group, we agreed upon a 3-step approach as an efficient and straightforward method for creating an MDT. Ultimately, we recommended that the formation of an MDT start with a single physician who has a passion for treating patients with TSC and is eager to liaise with patient families, organizations, and other physicians, and who can help drive the process. Second, a core team composed of individuals essential to the functioning of the clinic, such as a care coordinator and members of the core medical specialties, should be formed. Lastly, a full MDT can be developed by incorporating additional specialists identified through an established reference network with the assistance of the care coordinator. Based on our subsequent online correspondence and Delphi process, securing this care coordinator was considered the most important aspect of the MDT to implement. Other important factors for the MDT to incorporate included identifying a lead physician, the core group of physicians, and the team associated with the clinic. Informing treating physicians/family doctors/first-line health care workers is essential for a good patient follow-up, especially if the patient lives relatively far from the MDT. This team should also agree to follow established guidelines and agreed-upon protocols and work towards establishing transition of care as patients age [28, 29]. Others have published on establishing an MDT and noted similar features such as the specialists involved, a care coordinator, contact with primary care physicians, and including a patient database [29]; however, this prior report was descriptive of their personal experience as opposed to providing committee-based recommendations and including the patient’s perspective as in our research.

Currently, more literature regarding the benefits and goals of establishing a multidisciplinary care team in TSC is needed. This report discusses the steps to establish an MDT and our accompanying rationale. Future studies demonstrating the benefit of MDTs would be of value, particularly considering the benefits demonstrated from previous studies evaluating MDTs for the treatment of cancer [27]. One of the few available studies in TSC by Fujimoto et al. describes how the implementation of a TSC board to organize interdisciplinary management led to higher rates of regular follow-up, opportunities for patients to undergo examinations, opportunities for patients to receive neurologic treatment, and mTOR inhibitor usage compared with before the TSC board was implemented [31]. Studies of MDTs for TSC could also evaluate differences in treatment outcomes and patient satisfaction. Consensus recommendations do advocate for the efficient use of resources through the establishment of transition clinics where both children and adults can receive treatment. The aim of these clinics is to reduce duplicative tests and services while ensuring that appropriate surveillance and management are in place to prevent more costly medical complications and reduce mortality associated with TSC [19, 39]. Additional questions to be answered by future studies include what value certain specialties can add to an MDT, how they can best work with other parts of the health system (and education and social support system), and how they can contribute to improving long-term health and quality of life for patients with TSC and their families. Future studies should also focus on how to best provide a holistic approach to managing TSC, including such areas as education and psychosocial support.

From the perspective of health care providers, an MDT allows for an organized approach to tackling a multi-faceted disease. It enables improved management of care through establishing agreed-upon protocols, care coordinators and other points of contact, and modes of communication. The MDT also promotes communication between specialists to help ensure that all are aware of the patients’ co-existing conditions and medications and facilitates the transition from pediatric to adult care. Furthermore, the MDT is able to organize participation in clinical research and provide educational opportunities for health care professionals within the team.

For patients and their caregivers, the MDT can ease the burden of utilizing and coordinating care by providing a central point of contact for physician visits, imaging/testing, and patient/family education. For example, the MDT can coordinate physician and imaging visits such that for any imaging that requires sedation could also be used for concurrent eye and dental examinations, blood tests, or additional imaging studies (e.g., of the brain, kidney) that may be required. In addition, the team should be able to organize services to aid patients regarding particular aspects of TSC management, such as genetic counseling, transition of care services, mental health, and social support, and even provide on-the-scene observation, assessment, and intervention for TAND-related issues. Finally, the team should build a protocol that is adapted to the needs of patients with TSC, the facilities of the center, and available resources.

Our report has several limitations. While in our initial meeting we had planned to include patients/patient advocates in the development of our report, they were not included in the initial manuscript stages. However, the patients/patient advocates who were consulted contributed substantial feedback which greatly influenced our final report. In addition, the attendees of the initial meeting, which called for one expert for each specialty involved in TSC management, were specialists and thus the expertise presented comes primarily from the specialist perspective for practical reasons. No MDT formation specialists were consulted. We acknowledge that the composition of those who participated to this Delphi process and theoretical exercise do not necessarily represent the composition of the MDT itself and some participants in the management of TSC were not directly consulted. For example, while continuous and general care is performed by general pediatricians in children, general internal medicine in adults, general practitioners for all ages, these general practitioners were not included as part of the expert committee on TSC, though the coauthors do have experience as pediatricians and internal medicine physicians in addition to their specialties. Other potential participants in the management of TSC who were not consulted or discussed at length in this report include nurses, paramedics, psychologists/behavioral therapists, intellectual disabilities specialists, physiotherapists, communication specialists, behavioral therapists, and social workers. All of these care providers may not necessarily be a formal part of the MDT but play important roles in patient care. The authors also point out that our suggestions do not account for national or regional differences in care or discuss factors which may facilitate or limit formation of MDTs and recommend these areas for future study.

## Conclusions

The authors recommend a 3-step approach to building an MDT, starting with a single dedicated individual to begin organizing care, expanding to the development of a core team, and finally, establishing a full MDT. The authors recommend implementation of certain key aspects to create an effective MDT—most importantly, identifying a case manager to help coordinate care, providing access to health care professionals from a number of specialties, and including a lead physician who takes medical responsibility for the patients’ overall care.

## Abbreviations

MDT: Multidisciplinary team
MRI: Magnetic resonance imaging
mTOR: Mammalian target of rapamycin
TAND: TSC-associated neuropsychiatric disorder
TSC: Tuberous sclerosis complex
